# A Fully Atomistic Model of the Cx32 Connexon

**DOI:** 10.1371/journal.pone.0002614

**Published:** 2008-07-02

**Authors:** Sergio Pantano, Francesco Zonta, Fabio Mammano

**Affiliations:** 1 Institut Pasteur of Montevideo, Montevideo, Uruguay; 2 Venetian Institute of Molecular Medicine (VIMM), Padova, Italy; 3 Consorzio Nazionale Interuniversitario per le Scienze Fisiche della Materia (CNISM), Rome, Italy; 4 Dipartimento di Fisica “G.Galilei”, Università di Padova, Padova, Italy; Temasek Life Sciences Laboratory, Singapore

## Abstract

Connexins are plasma membrane proteins that associate in hexameric complexes to form channels named connexons. Two connexons in neighboring cells may dock to form a “gap junction” channel, i.e. an intercellular conduit that permits the direct exchange of solutes between the cytoplasm of adjacent cells and thus mediate cell–cell ion and metabolic signaling. The lack of high resolution data for connexon structures has hampered so far the study of the structure–function relationships that link molecular effects of disease–causing mutations with their observed phenotypes. Here we present a combination of modeling techniques and molecular dynamics (MD) to infer side chain positions starting from low resolution structures containing only Cα atoms. We validated this procedure on the structure of the KcsA potassium channel, which is solved at atomic resolution. We then produced a fully atomistic model of a homotypic Cx32 connexon starting from a published model of the Cα carbons arrangement for the connexin transmembrane helices, to which we added extracellular and cytoplasmic loops. To achieve structural relaxation within a realistic environment, we used MD simulations inserted in an explicit solvent–membrane context and we subsequently checked predictions of putative side chain positions and interactions in the Cx32 connexon against a vast body of experimental reports. Our results provide new mechanistic insights into the effects of numerous spontaneous mutations and their implication in connexin-related pathologies. This model constitutes a step forward towards a structurally detailed description of the gap junction architecture and provides a structural platform to plan new biochemical and biophysical experiments aimed at elucidating the structure of connexin channels and hemichannels.

## Introduction

Intercellular gap junction (IGJ) channels are ubiquitous components of higher organisms that permit the direct exchange of ions and molecules up to a molecular mass of ∼1 kDa between neighboring cells and thus play fundamental functions in intercellular communication between the vast majority of cell types (for comprehensive reviews, see [1], [2]). IGJ channels are formed by the end–to–end non-covalent docking of two hexameric oligomers, named hemichannels or connexons [3], each provided by one of the two neighboring cells. Each one of the six subunits, named connexins [4], in the annular assembly of a connexon, comprises four hydrophobic transmembrane (TM) segments, designated TM1 to TM4 [5]. N– and C–terminal tails and one connecting loop are found within the connexin cytoplasmic region, whereas the remaining two extracellular loops permit hemichannel docking and formation of a full intercellular channel that excludes the extracellular environment [3].

Over 20 different connexin genes have been identified in mouse and human genomes [6] and spontaneous mutations in these genes have been linked to the pathogenesis of several diseases, including disorders of the heart, skin, ear and lens [7]. In particular mutations of GJB1, the gene which encodes connexin 32 (Cx32), have been implicated in some forms of the X–linked Charcot–Marie–Tooth (CMTX) disease, an inherited sensory and motor neuropathy [8].

Although a considerable effort has been devoted to elucidating structural determinants and to clarify structure/function relationships of these channels, only medium− to low−resolution structures have been obtained so far (reviewed in [9]). Major contributions towards the structural determination of gap junctions have been provided by electron cryomicroscopy of channels formed by Cx43 [10]. Based on a more accurately resolved structure, Fleishman et al. [11] proposed a model for the arrangement of Cα carbon atoms in the TM helices which, owing to the wealth of data from patients with naturally occurring CMTX mutations, were mapped onto the amino acid sequence of Cx32. This choice is supported by the high degree of sequence homology in TM domains of different connexins, suggesting similar TM architecture [12]. However, it has been pointed out [9] that the structure proposed in ref. [11] may not represent a consensus model owing to disagreements in several experimental reports [13]–[16]. Indeed, corrections to the assignment of the helix orientations are expected due to the poor vertical resolution (∼2 nm) of the electron density map.

We have recently applied molecular modeling and simulation techniques to construct an atomic model of the TM part of a connexon, based on published Cα scaffold [11]. Despite the shortcomings due to carrying out molecular dynamics (MD) simulations in the absence of an explicit membrane environment, our TM model provided important structural insights into the effects of the point mutation methionine to threonine at position 34 of Cx26 (Cx26M34T) within the TM phase of the connexon. The model prediction that this mutation leads to a constriction of the channel pore accords with experimental data obtained by various techniques [17]. A similar model of the wild type Cx26 IGJ channel, combined with use of the Renkin's equation, predicted a value for the unitary permeability of Cx26 to cAMP that is in agreement with the experimental estimate obtained by a novel method based on FRET microscopy [18]. The application of our modeling procedure to the well-resolved structure of the potassium channel from *Streptomyces Lividans* (KcsA), taken as a control case (see Supporting Information Text S1), provided root mean fluctuations per residue of nearly 2 Å (see Supplementary Information, Figure S1), in line with the results obtained by previous theoretical studies within explicit membrane-like environment [19].

In this article, we report the construction of the first model of a full connexon within a phospholipid bilayer. Our primary aim was obviating limitations present in our original design that, in the absence of modeled extracellular and cytoplasmic loops, lacked connectivity between the TM helices. Prediction of the side chain positions allowed us to re–evaluate the relative orientation of the TM helices. Our calculations provide new molecular insights (*i*) on the role played by specific amino acids in keeping the correct folding of individual connexins and (*ii*) on the nature of the protein−protein interactions that hold together the quaternary structure of the connexon. These goals have been achieved by devising a practical protocol that combines modeling techniques with MD to produce interpretable protein models from a set of Cα coordinates corresponding to low resolution structures.

## Results

### MD drives the modeled system to a stable state

**Figure 1:**
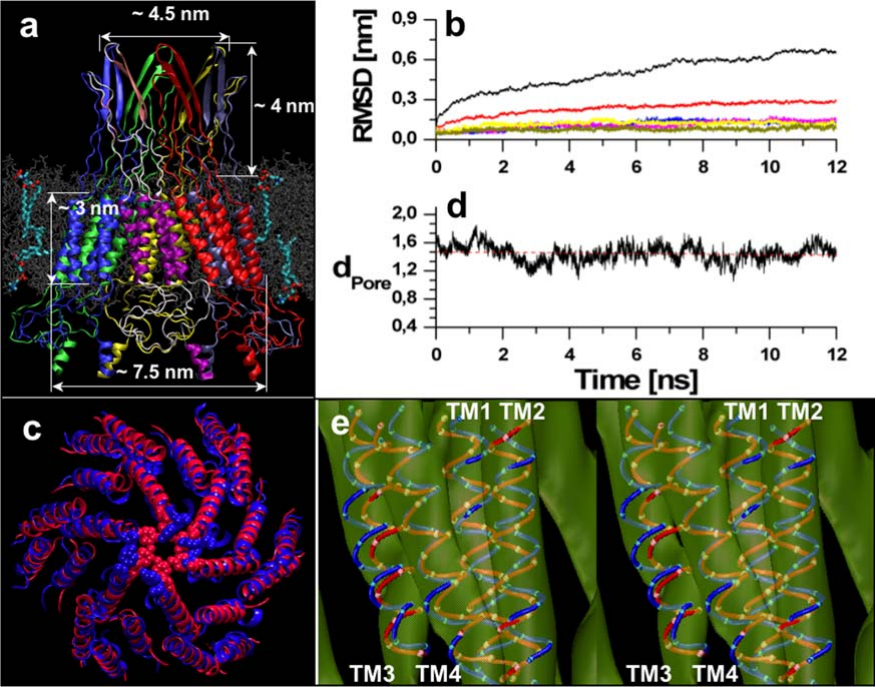
Overview of the connexon model in a membrane patch and dynamical behavior. a) Cartoon of the side view of the connexon. Each connexin is depicted with different colors. The central connexin is additionally colored by structure: pink for helices, white for coils and light pink for β–strands. The phospholipid membrane, embedding the hemi–channel, is represented in gray. Four phospholipids randomly chosen are colored by atom. b) Root mean square deviations (RMSD) calculated over the Cα atoms of the whole connexon (black) and over the sole TM domain of the connexon (red). Individual RMSD traces of each of the six connexin TM domains are practically indistinguishable and remain below 0.17 nm (lower traces). c) Structural superposition between initial (red) and final (blue) conformers. The Tyr151 residues that delineate the maximum constriction point of the channel pore are shown in space filling mode. d) Minimum diameter of the pore measured as the distance between the OH atoms of opposite Tyr151 in different connexins, averaged over the three possible pairs. The nearly horizontal red dotted line corresponds to a linear fit over the entire simulation, with slope of −0.004 and an y-ordinate of 1.45 nm. e) Stereo view of the rigid docking of the Cα models by Fleishman et al. (red tube) and ours (blue tube) onto the electron microcopy map of Cx43 for the TM part of one connexin seen from the membrane side. Cα atoms are also shown.

By construction, the overall structural determinants of the connexon (Figure 1a) meet those of recombinant, C–terminal truncated, Cx43 channels obtained by cryo–electron microscopy [10], [11]. Molecular mechanics protocols lead to conformations corresponding to local energy minima in the proximity of the starting configuration. We opted instead for MD simulations since temperature effects intrinsic in this technique avoid trapping by shallow local minima, and thus permit the entire complex to explore better the energy landscape. In the course of the MD simulations performed on the Cx32 model, the TM region featured RMSD deviations from the initial conformation ranging from 0.1 up to 0.2 nm for individual connexins, indicative of a very stable behavior. RMSD values for the whole connexin stabilized around 0.3 nm after a simulation time of nearly 5 ns (Figure 1b). The differences between the RMSD calculated over the TM domain of the connexon and those of the separated connexins indicates that the structure of the helix bundles remains stable while global quaternary structure rearrangements take place in the course of the simulation. The observed dynamical behavior is in agreement with a general analysis of the convergence achieved by MD simulations of different membrane proteins within a time window of the order of 10 ns [20].

### Pore size

The reported maximum constriction point of the gap junction sits within the TM domain [10]. Consistently, the pore diameter predicted by our model has its narrowest point at the level of Tyr151 (Figure 1c), resulting in an average internal diameter, d_Pore_, calculated between opposite OH moieties that fluctuated during the MD simulation from 1.1 to 1.7 nm. A linear fit gives a nearly horizontal line (slope −0.004) at d_Pore_ = 1.4 nm, underlining the good convergence for the TM domain (Figure 1d). Considering intrinsic fluctuations, this value agrees well with the range from ∼1.1 nm estimated by perfusion of polyethylene glycol probes [21] to ∼1.5 nm estimated by [9] based on cryoelectron microscopy data [10]. Our results point to a highly mobile behavior of the channel, originating from quaternary structure oscillations as well as side chain mobility of the single residues. Indeed, if an average d_Pore_ size of ∼1.4 nm is assumed, fluctuations are needed to account for the passage of larger fluorescent tracers such as Alexa Fluor594 [22] that have a minimum (non solvated) diameter of nearly 1.6 nm. Thus, our simulations suggest that large permeating molecules may slowly creep through gap–junction channels exploiting positional rearrangements of the connexin subunits powered by thermal energy. In so doing, they unavoidably interact with pore residues. These interactions, together with geometrical constraints, will therefore be the key determinants of the molecular permeability to larger solutes, including key metabolites and second messengers [18].

### Comparison with electron microscopy map

Aimed to compare the gross geometrical determinants of our model against the raw experimental data we performed a rigid fit of the Cα atoms of the final (averaged, see Methods section) connexon structure, using the same cryoelectron microscopy data of ref. [11]. Small deviations were found respect to the original model in the positions of TM3, which slightly moved towards the pore lumen (Figure 1e). The mismatch in the pitch of helix TM2 is described in the next paragraph. Although the map is too coarse to permit meaningful quantitative comparisons, visual inspection of the best fits indicates that both models match equally well the experimental data (Figure 1e).

### Orientation and structure of the TM2 helix

**Figure 2:**
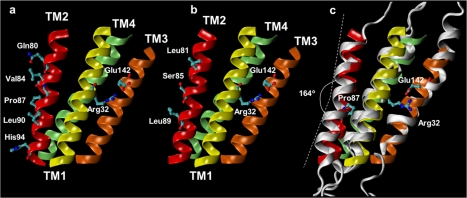
Side view of the connexin four–helix bundle for different orientations of the TM2 helix. a) TM2 (red) oriented as proposed in [11]. The ribbons representing the other three TM helices are colored in yellow (TM1), green (TM3) and orange (TM4). TM2 residues involved in CMTX disease, according to the Swiss–Prot database (entry code P08034), are represented with sticks. Residues Arg32 (TM1) and Glu142 (TM3), which form a salt bridge exposed to the pore lumen are shown for reference. b) Same as (a) but with the TM2 orientation proposed in this work. c) RMSD fit between the initial and average conformation during the last 5 ns of simulation (white). White dashed lines highlight the kink introduced by Pro87 in TM2.

In the original assignment by Fleishman and coworkers [11], the relative rotation angles of the α helices fitted into the density map were estimated by analysis of evolutionary conservation and hydrophobicity of amino-acid residues. Thus TM2 in ref. [11] occupies the most peripheral position within each of the six connexins, facing the membrane lipid phase, and consequently it is the only helix not involved in inter−connexin interactions. However, 6 out of 13 TM2 residues reported to be involved in CMTX disease (see www.expasy.org/uniprot/P08034 and linked refs. therein) are exposed to the hydrophobic phase, suggesting that an alternative orientation is possible. Therefore, we sought to explore different helical orientations that would increase the contact area between TM2 and the rest of the four helix bundle, and reduce the inter−helical gap volume (Table 1). Using rigid docking techniques, we obtained a different orientation for TM2, rotated nearly 180 degrees respect to ref. [11], which presents a higher degree of surface complementarity. In our model only 3 residues involved in CMTX disease, namely, Leu81, Ser85 and Leu89 face the lipid phase, and thus, do not contribute to the formation of inter helical contacts. Figure 2a,b show a comparison between TM2 in our structure and that of ref. [11].

**Table 1:** Surface complementarities between different orientations of TM2.

	TM 1, 3, 4	TM 2
**Model A**		
Interface ASA^a^	712.4	769.6
% of interface ASA	13.1	31.7
GAP volume index^b^	1.5	
**Model B**		
Interface ASA	701	832,9
% of interface ASA	13	34.2
GAP volume index	1.2	

The model as obtained from the original Cα coordinates ([11] PBD entry code 1TXH) and the one obtained in this work are referred as Model A and B respectively.

a Accessible surface area.

b Defined as = Gap Volume/Interface ASA

This new orientation proposed for TM2 is supported by the exhaustive analysis of disease-causing mutations within the segment from Arg75 to Val95, both in Cx32 and in its highly homologous isoform Cx26 (see Discussion).

Although the low resolution map does not show any obvious kinks in the TM domain, residues Leu83 through Pro87 defined a “proline kink” (PK) segment in a model of isolated TM2, where hydrogen bonding of Thr86 to the backbone carbonyl of Ile82 causes a 37 degrees bending of the helix that corresponds functionally to the channel open state [23]. The model proposed in ref. [11] was constructed by fitting canonical, i.e. straight, α−helices to the map in ref. [10] and thus shows no sign of a PK in TM2. MD relaxation in our model retrieved the PK early in the simulation, and stabilized around an average bend angle of 16±5 degrees (mean±S.D.) (Figure 2c). This more conservative estimate arises as a consequence of the constraints imparted to TM2 by its protein and membrane environment. Obviously, these constraints were not present in the isolated TM2 model [23]. Along the MD trajectory, we observed the formation of H−bond interactions between the Thr86 and the backbone carbonyl of Ile82, as well as between Ser85 and the backbone carbonyl of Leu81 (see Figure S2a), confirming their stabilizing role in TM2 bending.

### Main determinants of connexon stability associated to electrostatic interactions

Inspection of the MD simulation trajectory reveals the formation of four salt bridges within each single connexin chain. Since energy gain upon formation of solvent−exposed charged groups is rather small [24], [25], it is expected that these interactions may break and form dynamically due to competition with the solvent. Nevertheless, bond occurrence was characterized by relatively prolonged time intervals (Table 2), indicating that salt bridges may play an important role in stabilizing the connexins fold.

**Table 2:** Selected salt bridge interactions calculated over the 12 ns of MD simulation (see also figure 3).

H Donor– Acceptor	Occupancy [%]
**Arg22_(TM1)_–Glu208_(TM4)_**	**65**
**Arg32_(TM1)_–Glu146_(TM3)_**	**87**
**Arg75_(TM2)_–Glu41_(TM1)_**	**53**
**Arg75_(TM2)_–Glu186_(TM4)_**	**51**
**Lys187_(TM4)_–Glu186_(TM4)_**	**50**

**Figure 3:**
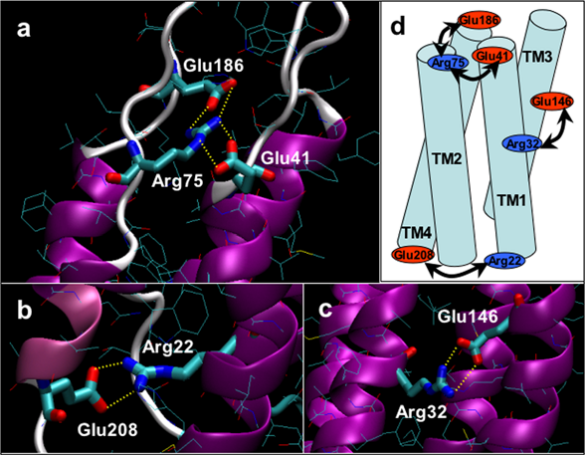
Main electrostatic interactions from representative snapshots of the MD simulation. a) Salt bridge network between Arg75 (TM2) and Glu41 (near TM1)–Glu186 (near TM4) located at the outer leaflet of the plasma membrane. b) Interaction between Arg22 (TM1)– Glu208 (TM4) at the inner leaflet of the membrane. c) Salt bridge formed by Arg32 (TM1) and Glu146 (TM3), located nearly in the middle of the membrane and facing the pore lumen. d) The scheme highlights the putative influence of above interaction in keeping the folding of the connexin.

The first two salt–bridges are formed between Arg75 (TM2) with Glu residues at the extracellular side (Figure 3a), namely Glu41 (TM1) and Glu186 (TM4). The occurrence of these interactions accords with the complete connexon dissociation observed upon solubilization in dodecyl maltoside for the Cx26R75D and Cx26R75W mutants [26]. In apparent controversy, the Cx26R75A mutant seems to fold correctly and exhibits detergent resistance [26]. A possible explanation for this behavior is the presence of neighboring Lys187. During our simulations, this residue interacted with solvent/phospholipid molecules, but also established fluctuating salt bridges with Glu186 (Table 2). It can therefore be speculated that the stabilizing effect of the salt bridges between Arg75 and Glu186/Glu41 can be partially rescued by Lys187 in the presence of a small residue such as Ala that may confer local structural flexibility. Furthermore, connexons affected by the Cx26R75W mutation are sufficiently stable to form functional hemichannels in membranes although their properties differ from those of wild type Cx26 hemichannels, thus the absence of gap-junctional communication imparted by this mutation is not due to lack of connexon stability [27] but may result from confined structural distortions due to missing electrostatic stabilization between Glu41 and Glu186. This may destabilize docking between apposed connexons, resulting in defective gap junction channels.

The third salt bridge is formed between Arg22 (TM1) and Glu208 (TM4), at the cytoplasmic side (Figure 3b). This interaction, which is present in more than 50% of the simulation is consistent with R22G and R22P being missense mutations [28].

The fourth salt bridge between Arg32 (TM1) and Glu146 (TM3) occurs midway through the membrane, exposed to the pore lumen (Figure 3c). The formation of these two salt–bridges has been recently validated by double compensatory mutants (K22E, E209R and R32E, E146R) [29]. Hence these strong inter-helix electrostatic interactions may contribute to tie together helices TM1 to TM4 (Figure 3d).

### Hydrophobic interactions

**Figure 4:**
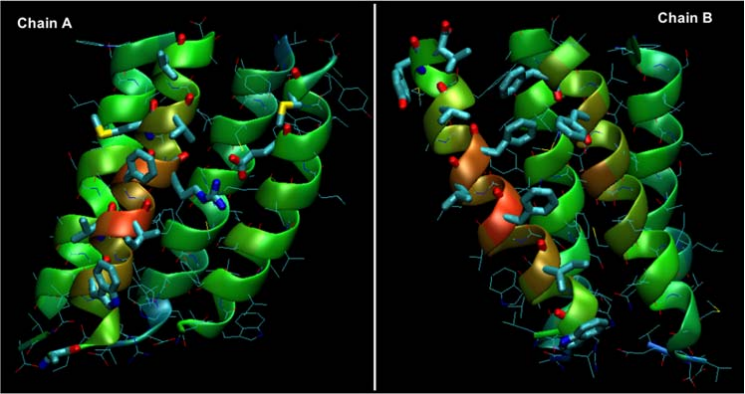
Inter connexin surface. Open-book representation of the average connexin dimer. The thick residues are those involved in protein–protein interactions reported in Table 3. Ribbons are colored by position going from red, to green and to blue from the center of the dimer.

As shown in Figure 4, contacts between adjacent connexins, named *A* and *B*, are mediated by helices TM1 and TM3 in *A*, and by TM3 and TM4 in *B*. As previously observed, TM2 is the only helix that does not participate in inter−connexin contacts. In our simulations, these were essentially of hydrophobic nature and no firmly established electrostatic interactions were detected between adjacent connexins within the TM region. The average interface area between connexon subunits is 540 Å^2^, with 15 % and 7 % of polar atoms in the interfaces of connexin subunits *A* and *B*, respectively. Notably, several aromatic residues are located within the interface (two in connexin *A* and 5 in connexin *B*, Table 3), which could further strengthen the quaternary complex through aromatic−aromatic stacking interactions.

**Table 3:** Residues belonging to the TM region of the inter−connexin interface.

Residue _(Helix)_	Interface ASA	% Interface ASA
Connexin ***A***		
ALA 19 _(TM1)_	35.5	6.5
TRP 24 _(TM1)_	86.9	16
VAL 27 _(TM1)_	15.6	2.9
ILE 28 _(TM1)_	30.1	5.5
PHE 31 _(TM1)_	113.4	20.8
ARG 32 _(TM1)_	48.5	8.9
MET 34 _(TM1)_	33.7	6.2
VAL 35 _(TM1)_	39.9	7.3
VAL 38 _(TM1)_	61.8	11.4
GLU 146 _(TM3)_	10.7	2
MET 150 _(TM3)_	61.6	11.3
Connexin ***B***		
TRP 133 _(TM3)_	40.6	7.7
ILE 137 _(TM3)_	41.6	7.9
PHE 141 _(TM3)_	79.9	15.1
LEU 144 _(TM3)_	84.3	15.9
PHE 145 _(TM3)_	22.3	4.2
VAL 148 _(TM3)_	41.8	7.9
TYR 151 _(TM3)_	63.3	12
VAL 152 _(TM3)_	32.4	6.1
PHE 190 _(TM4)_	54.6	10.3
PHE 193 _(TM4)_	65.2	12.3

Accessible surface area (ASA) is given in Å^2^ units. See also figure 4

### Covariance matrix

**Figure 5:**
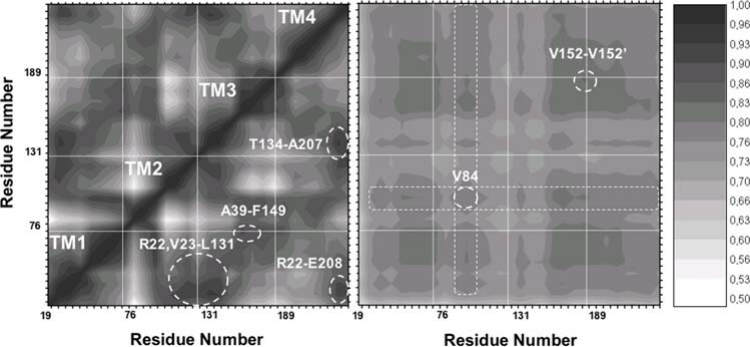
Normalized covariance matrix averaged over the six TM domains. Dark and light regions correspond to high and low correlation values respectively. White lines indicate the separation between helices. Numbers on the axes indicate the first residue of each of the TM helices, ticks are drawn every two residues. Major correlation islands commented in the text are indicated with dotted circles. To avoid redundancy, the blocks corresponding to interactions within four helices of one single connexin (a) and to interactions of helices belonging to adjacent connexins (b) are presented.

Given the potential relevance of the structural fluctuations, we turned our attention to the study of the normalized covariance matrix (CM) along the MD trajectory. This study may reveal the occurrence of correlated motions between segments of the proteins that are not directly linked [30]. The normalized CM is symmetrical and ranges from −1 to 1 for anti correlated and fully correlated motions, respectively. To ease data interpretation, we calculated the CM over the TM domain. Furthermore, we reduced the redundancy in the representation by averaging over each connexin (Figure 5a) and over each connexin dimer (Figure 5b). This allowed us to examine, respectively, interactions within individual connexins and inter−connexin interactions between adjacent proteins. The diagonal values of the matrix in Figure 5a correspond to autocorrelation, which is obviously unitary, whereas the off−diagonal elements gauge interactions between distinct residues of the same connexin protein. The averaged CM values range from 0.45 to 1, indicating highly correlated motions. However, correlation is higher among TM1, 3 and 4, whereas the kink imparted by Pro87 to TM2 (see above) reduces correlation. The lowest degree of correlation occurs at Trp77, whose bulky side chain is completely exposed to the exterior of the connexon (Figure S2b). Correlation peaks indicate strong interactions among residues reported to be involved in CMTX, namely: Arg22, Ser26 and Phe29 (TM1) with Trp132, Thr134 (TM3) and His94 (TM2), all located at the cytoplasmic side. Other important correlation peaks signal interactions of Thr134 (TM3) with Ala207 (TM4) and Ala39 (TM1) with Phe149 (TM3). Of notice, mutations A39P, A39V and F149I are involved in CMTX [31]. Finally, Arg22 (TM1) and Glu208 (TM4) are correlated by salt bridge interactions (Figure 3b).

The generally lower CM values in Figure 5b indicate weaker dimeric interactions. The largest peak is centered on Val152, at the extracellular end of TM3, which is a contact point with Val38 and, to a lesser extent, with Val35 in the TM1 of the adjacent connexin. Similarly, the quaternary assembly produces islands of high correlation between the C–terminal parts of TM1, TM3 and the N–terminals of TM4 (Figure 5b). The region surrounding the mid point of TM2 shows a less obvious behavior. The broad maximum is centered on Val84, which shows remarkable spatial covariance with residues belonging to other helices of contiguous protein subunits, with peaks at Phe31 (TM1), Tyr135 (TM3), Phe145 (TM3) and Val152 (TM3), despite the lack of direct contacts. This outcome accords with subtle mutations like Cx32V84I [31] and Cx26V84L [32] imparting a pathological phenotypes without major alteration in the unitary ionic conductance [33].

## Discussion

Reaching near−atomic resolution for the structure of biologically important macromolecules, such as integral membrane proteins, is still a challenging task despite progress in specimen−preparation techniques. In the case of connexins, breaching the resolution limits of 5−10 Å is likely to require several years. In this interim period, molecular simulations substantiated by the wealth of molecular biology data existing in the literature may provide valuable tools [34] that, akin to a computational nanoscope, permit to derive images of complex and otherwise inaccessible biomolecular systems.

The structural model presented here is the first attempt to provide an atomistic view for the TM domain of a connexon assembly within a realistic membrane, solvent and ionic environment. Our model, which relies on the assignment of the Cα scaffold into a low resolution map, is in agreement with a variety of experimental data derived from mutagenesis analyses, as discussed below, although small deviations from the real structure cannot be excluded at present. Comparison with the test case of the KcsA potassium channel suggest that possible errors associated to our assignments are upper bounded by the dimensions of single amino acids (see Supporting Information Text S1).

Different assignments of the hydrophobic domains M1–M4 to the four helices in the cryo-EM map have been proposed [35]. Among them (reviewed in Kovacs et al., [36]), experimental data provide strong support for the positioning of TM3 and TM4 proposed in ref. [11]. That configuration is also consistent with TM1 forming a salt bridge between its Arg32 and Glu146 in TM3 (of the same connexin) [29]. However, data regarding the orientation of TM2 are of far less straightforward interpretation. Indeed, in contrast to M3 and M4, the residues in M1 and M2 are homogeneously conserved (as noted in the Figure 2a of ref. [11]) so that the orientation around their principal axis cannot be determined reliably on the basis of conservation alone. Our choice for the TM2 orientation is based on the analysis of disease−associated mutants and polymorfisms of Cx32, and the closely related Cx26 (see http://davinci.crg.es/deafness/, http://webh01.ua.ac.be/hhh/, http://www.molgen.ua.ac.be/CMTMutations/ and at NCBI's Online Mendelian Inheritance in Man server http://www.ncbi.nlm.nih.gov/entrez/query.fcgi?CMD=search&DB=omim). Of notice, the TM2 of Cx32 and Cx26 differ by only two amino acids: Ser78 and Leu83 in Cx32 which are respectively replaced by Ala78 and Phe83 in Cx26. Chimerical Cx32 channels, obtained by substitution of a mutant TM2 from Cx26, show that this helix plays a critical role and is involved in voltage gating [23]. Hereafter we summarize the salient features of our analysis.

Mutation of Arg75 to Trp in Cx32 (Cx32R75W), at the junction between the first extracellular loop (E1) and TM2, has twice been implicated in X–linked Charcot–Marie–Tooth disease [37], [38]. The corresponding mutation, Cx26R75W, has been found in patients with dominant autosomal deafness and palmoplantar keratoderma [39]. No other dominant mutations are found along the TM2 helix of Cx26. It has been suggested that the highly non−conservative amino acid substitution of a Trp into the interface between the α−helical TM2 region and the rigid β−sheet forming E1 is likely to alter the secondary structure of the protein [40]. However, functional studies have recently determined that the absence of gap−junctional communication is due to defects in the assembly of mutant hemichannels into full gap−junction channels, whereas the hemichannels *per se* retain partial channel activity when examined in the unpaired configuration [27]. Consistent with these findings, TM2 in our model has Arg75 pointing towards the interior of the connexin four−helix bundle, in good position to form a double salt bridge interaction with Glu41 and Glu186 (Figure S2c). Thus we conclude that neutralization of the positive charge at position 75 generates important rearrangements at the extracellular side of the TM segment, near the maximum constriction zone of the channel [10].

Proceeding along TM2 we meet Trp77. The recessive mutation Cx26W77R causes deafness [41] and prevents the formation of homotypic junctional channels in heterologous expression systems [42]. Accordingly, of the 48 cysteine substitutions made at Cx32 only W77C and two others (W133C, and T134C) resulted in nonfunctional channels [13]. We can rationalize these outcomes by noting that this is the only Trp near the extracellular membrane leaflet (Figure S2b) and, in our model, it lies in good position to establish specific interactions with cholesterol molecules, which may play a role in connexins trafficking or plasma membrane localization [43].

Although modification of several residues in TM2 may critically affect the permeability properties of the channel, this is not the case for Leu83 in Cx32 and the corresponding residue, Phe83, in Cx26. Indeed, Cx26F83L is a polymorphism [42]. In our model Leu83 is packed against Ser198 (TM4), which is frequently substituted among different connexin isoforms by other small residues, such as Ala, leaving enough space to accommodate the L83F mutation. Indeed, replacement of the Phe side chain in our model does not generate steric contacts at all, consistent with the lack of phenotypes for this substitution (Figure S2d).

In stark contrast, the next residue in the line, Val84, is invariant in all connexins. Cx32V84I is a physicochemically moderate mutation that was found to cause CMTX disease [44] (see also Supplementary Table 2 at http://bioinfo.tau.ac.il/~sarel/GJ.html,) whereas Cx26V84L is a rare mutation associated to nonsindromic deafness [32]. It leaves the unitary ionic conductance of homotypic Cx26 channels unaffected but has a deep impact on the permeability to InsP_3_ [33], a second messenger that plays numerous key roles in Ca^2+^ homeostasis. Leu is slightly bulkier than Val, and our model places Val84 facing the helix bundle in close contact with Met34 (TM1). Mutations of Met34 to smaller residues (Ala, Thr) lead to a progressive reduction of unitary conductance [13]. We have recently shown that Cx26M34T is a pathogenic mutation implicated in hereditary deafness [17]. Hence we speculate that, in a sort of domino effect, substitution of Val84 by bulkier residues, such as Leu or Ile, may distort the tertiary structure through clashes with Met34, forcing TM1 to further expose its positively charged Arg32 into the pore lumen (Figure S2e). This would modify the possible interaction of Arg32 with negatively charged InsP_3_ molecules traversing the pore, ensuing in a reduced unitary permeability for this second messenger. Replacing Val84 with the smaller Ala should not trigger these changes. Indeed, the related mutation Cx26V84A is classified as a polymorphism [45], consistent with this conclusion.

Mutations in the next two polar residues, Ser85 and Thr86, have been reported to reduce open channel probability at null junctional potential, *V_j_* = 0 (Cx32S85C, [46], [47] and Cx32T86A/V/L/N, [23]). For Thr86, this outcome has been interpreted as due to the loss of hydrogen bonding to the backbone carbonyl of Ile82 [23]. In our model, Ser85 and Thr86 point towards the lipid phase and form H−bonds with the carbonyl oxygen of, respectively, Leu81 and Ile82 one helix turn above. Similar features were detected in the course of our MD simulation (Figure S2a). Interestingly, early compilations of the Cx26 sequence reported a Ser at codon 86 [48], see also: http://www.ncbi.nlm.nih.gov/entrez/viewer.fcgi?val=AAD21314.1), implying that T86S, which maintains intact the H-bonding capabilities, is simply a polymorphism.

Next, mutation Cx32P87A is yet another cause of the CMTX disease; nonetheless it forms functional channels with properties that differ mildly from those of wild−type controls when expressed in pairs of Xenopus oocytes [23], [49]. The subtle effects of P87A suggest that the phenotype is caused by impaired exchange of solutes larger than simple ions [50]. Furthermore, exchanging Pro with Val rather than Ala at codon 87 prevents expression of junctional currents in oocytes, suggesting that a progressive raise in the side chain bulkiness at position 87 may cause increasing levels of distortion in the tertiary and/or quaternary structure. However, the conformations of Val in α−helical environments are restricted to one rotamer population due to steric hindrances with the backbone of the preceding turn [51]; hence, an alternative possibility is that the conformational restriction of the Val side chain imparts local rigidity to the α−helix.

Pathological effects have been reported for other residues of Cx26 along TM2, with a periodicity of nearly 3−4 residues (see http://ca.expasy.org/uniprot/P29033). Thus, similar to Cx26V84L [33], the deafness−linked Cx26A88S mutation [52] specifically affects the intercellular exchange of larger molecules but leaves the ionic permeability intact [53]. Two other related mutations, Cx30A88V [54] and Cx32A88V [55] are respectively associated with hydrotic ectodermal displasia, a skin disorder, and CMTX.

Mutation of the next residue, Leu89, to Ile (as in Cx26L89I) results in simple polymorphisms without known deleterious effects [56], in agreement with its orientation towards the lipid phase (Figure S2f). However, Cx32L89P has been reported to be related with inherited peripheral neuropathies [31]; indeed Leu substitution with Pro is more severe, having the potential to alter the helical structure of TM2.

Mutations at Leu90 are associated to CMTX in Cx32 [31], and to hearing loss in Cx26 varying from moderate to profound [57], [58]. In expression studies, Cx26L90P proteins were detected by immunofluorescence at contacting HeLa cell plasma membranes [59] but failed to induce the formation of homotypic channels [42]. The same fate occurred to Cx32L90H [50]. Cx26L90P mutant channels inserted into the plasma membrane of oocytes at a degree similar to wild type Cx26 but failed to induce any measurable macroscopic current; yet, co−expression of mutant and wild type channels, resembling the situation of heterozygous individuals, did not significantly affect the electrophysiological properties of the wild type Cx26, consistent with a recessive phenotype [60]. In summary, these results indicate that the above mutations at codon 90 do not prevent protein targeting to the plasma membrane, but severely compromise homotypic channel function. In our model Leu90 is packed within the hydrophobic environment of the four helix bundle (Figure S2g). Thus the introduction of a polar residue, as in the L90H mutation, may significantly distort the tertiary structure of individual connexins. As observed above, important changes to the secondary structure of TM2 are also expected in the L90P mutation due to the introduction of Pro that may destabilize the helical conformation near the cytoplasmic mouth of the pore.

The V95M mutation affects similarly both Cx32 [50] and Cx26 [33] producing channels that insert in the plasma membrane but are electrically silent. V95M occurs at the interface of TM2 with the cytoplasmic loop, which is thought to be an important determinant of pH sensitivity. The previous residue, His94, also belongs to this interface and, consistently, Cx32H94Y mutants did not induce measurable conductance when paired in the homotypic configuration. This outcome has been ascribed to the high probability for at least one of the two hemichannels of being closed regardless of the transjunctional voltage [47]. Interestingly, the Cx32H94Q mutation produces functional channels that differ from wild type Cx32 only in the kinetics of the currents evoked by transjunctional voltage steps [47]. In agreement with this result, our orientation of TM2 packs both His94 and Val95 inside the helix bundle. Substitution of the bulkier Tyr at position 94 on the final averaged structure leads to important clashes. Instead no structural perturbation results from Gln replacing His at codon 94, which retains H−bonding capabilities (Figure S2h). Similarly, the longer side chain of Met at position 95 could push TM1 towards the channel lumen, obstructing the pore.

In summary, although the low resolution of the electron microscopy data hinder the possibility of a definitive assignment for TM2 orientation, the one proposed here is supported by extensive mutational analysis, providing putative structure-based rationales for the effects of 16 mutants/polymorphisms along TM2 reported in nearly 30 independently published papers based on a variety of experimental techniques. More detailed structural determination or ad hoc molecular biology experiments are required to definitively solve this issue.

We hope that the model presented here may constitute a structural scaffold to rationalize also the effects of other point mutations leading to pathological phenotypes. Furthermore, this theoretical framework, or possible future refinements, may aid the design of new experiments aimed at elucidating the structure/function relationships in gap junction channels. It may additionally guide the design of novel mutants with impaired conducting or folding capabilities as a tool for selectively knocking down or knocking out connexin function. Possible extensions concern comparative modeling studies aimed at clarifying the determinants of differences among connexin isoforms.

## Methods

### Structural model of the TM section of Cx32

As mentioned above, the atomic model of the TM portion of Cx32 was constructed on the basis of a Cα model of mouse Cx32 [11] (PDB entry code: 1TXH). The Cartesian coordinates of the remaining atoms of each residue were added in their canonical conformation using the XLEAP module of AMBER 8 [61]. This procedure generated a fully atomistic model of the TM portion of Cx32 affected by a number of steric clashes and internal tensions, corresponding to a high energy state. In this configuration, the system has a marked tendency to explode driven by van der Waals repulsions among the newly introduced side chain atoms. To overcome this problem, we devised a protocol based on MD, which allows the side chains to explore the available conformational space satisfying the restrictions imposed by the protein environment.

### Structural relaxation

Starting from the initial, unstable, set of all-atoms coordinates, the system underwent 20 ps of unconstrained MD, yielding large geometrical distortions. To recover the original Cα coordinates while simultaneously finding new positions for the side chain atoms, compatible with a low energy state for the whole system, we carried out three successive rounds of constrained MD of 100 ps each, in some analogy to a simulated annealing process. Thus the TM Cα carbons were pulled back towards their original positions by harmonic constraints whose stiffness constants were set to 0.1, 1 and 10 [kcal/mol−Å^2^] in the first, second and third round, respectively. Thereafter the system underwent full energy minimization (EM), first in the presence of constraints and then unconstrained. Throughout this initial stage, EM and MD simulations were performed in the presence of implicit solvent with the Generalized Born approach [62] implemented in AMBER 8 using the Amber94 force field [63] with the following settings: cutoff distance, 1.8 nm; time–step, 2 fs; salt concentration, 0.15 M; dielectric constant, 78; temperature, 100 K using Langevin dynamics with a collision frequency of 2 ps^−1^. The *Shake* algorithm was used to constraint all chemical bonds [64].

The reliability of this procedure was verified on the structure of the Potassium channel (KcsA) from *Streptomyces Lividans*, one of the best characterized ionic channels, solved by X-ray crystallography ([65], PDB entry code:1BL8). To this end, we used the protocol outlined above to attempt the reconstruction of the all-atoms structure starting from the sole Cα positions (see Supplementary Information). The resulting model yielded very excellent agreement, with a root mean square deviation calculated over all the heavy atoms below 2,5 Å and root mean square fluctuations of the side chains of 2,1 Å (s.d. 0,7 Å) (see Figure S1), i.e., values comparable to standard MD simulations using well resolved structures.

A comment is in order on the fact that the reliability of the above described procedure may depend strongly on the initial coordinates of the Cα atoms fitted into the low resolution electronic map. Therefore after initial relaxation of the atomistic model of the connexon we sought to reassess the positions of the TM helices within the connexin bundle.

### Re−evaluation of the TM2 packing within the helix bundle

Since the overall reciprocal orientation of helices TM1, 3 and 4 has strong experimental support, we reassessed TM2 orientation based on a criterion that maximized surface complementarities after the initial MD relaxation. To this end we performed rigid docking of TM2 onto the all-atoms scaffold provided by helices TM1, TM3 and TM4 using the program 3D–Dock [66]. Among the several configurations sampled, we adopted the solution with the highest surface complementarity and appropriate orientation relative to the membrane plane. The results corresponds to a ∼180 degrees rotation of TM2 about the helix axis relative to the configuration reported in ref. [11].

### Construction of the hydrophilic loops

Having achieved a stable structure for the TM domain, we undertook the construction of the remaining part of the connexon. In each connexin, the N–terminal residues from 1 to 10 were modeled as α–helices, based on NMR results derived from Cx26 fragments [67]. Residues 49 to 56 and 61 to 67, belonging to the first extra cellular loop, and residues 166 to 170 and 177 to 181, belonging to the second extra cellular loop, were modeled as double layered β−strands [68]. The C−terminal loop was truncated at Ala221 in accord with the deletion mutant used for the structural studies performed by [10]. The remaining residues for which no structural information is available were generated using the loop refinement option of the program Modeller 8.0 [69], [70] while keeping the TM domain spatially constrained. It must be noticed that, although an effort has been made to stick to the fragmentary structural data available, the starting conformation used for the hydrophilic loops correspond, at most, to an energetically *reasonable* conformation. Indeed, loops have been included for the sole purpose of providing a rough estimate of the elastic coupling, viscosity and long–range electrostatic contribution of their respective charged residues to the structure and dynamics of the TM domain.

### MD simulations in a membrane environment

The model of the full Cx32 connexon was inserted in a pre–stabilized membrane patch of palmytoil phosphatidyl choline (POPC) phospholipids; see reference [71] for details on the parameterization and stabilization of the bilayer. Phospholipid molecules within 0.2 nm of any protein atom were removed (173 in total). The protein–membrane system was solvated with 36699 TIP3P water molecules generating a simulation box of 11.7 nm×11.7 nm×12 nm. Rectangular periodic boundary conditions were used throughout. These dimensions yielded a distance between consecutive connexon images on the membrane plane comparable to that observed in real connexon plaques measured by atomic force microscopy [72]. Twenty-nine neutralizing Cl^−^ counterions and fifteen K^+^ Cl^−^ pairs were added to the solution, yielding an approximately physiological salt concentration. The whole system underwent energy minimization and 1.7 ns of MD stabilization. Temperature and pressure were kept constant using Berendsen's thermostat and barostat [73] at 300 K and 1 atm, respectively. Particle Mesh Ewald summation [74] was used for long range electrostatic interactions with a cut off of 0.8 nm for the direct interactions. Loose harmonic constraints of 0.1 kcal/mol−Å^2^ were applied to the N and P atoms of the phospholipids heads during the first 0.2 ns. Harmonic constraints of 0.5 kcal/mol−Å^2^ were applied to the Cα atoms of the TM domain of the proteins to avoid spurious distortions during the initial thermalization and pressurization of the system. Thereafter these constraints were linearly reduced to zero during the first 0.5 ns. Then, 1 ns of unconstrained MD was performed to fully stabilize the system. Finally, 12 ns of MD were carried out and used for analysis.

### Calculated properties

The TM domain of Cx32 was defined as the collection of the following segments: residues from 19 to 40, 76 to 96, 131 to 152 and 189 to 209. Positional root mean square deviations (RMSD) were calculated on the Cα atoms from the starting configuration of the solvated system. The salt bridges reported in Table 1 were considered within a cutoff distance of 0.35 nm between acceptor and donor and 120 degrees for the donor–H–acceptor angle. Since the time–evolution of each connexin is not constrained by symmetry operations, small structural and dynamic deviations are expected from one polypeptide chain to another. However, since all proteins composing the channel are indistinguishable, quantities reported in this article were calculated as averages over the MD trajectory and over the six proteins. The structure regarded as “final” was calculated as the time average over the last 5 ns of the MD trajectories and as the spatial average over the TM region of the six connexins. The structure resulting from the averaging was optimized by EM. A similar procedure was applied to derive an average connexin dimer, which was used to calculate the interface residues between contiguous connexins at the Protein–Protein interaction server available at http://www.biochem.ucl.ac.uk/bsm/PP/server/index.html. The normalized covariance matrix of the Cα atoms in the TM domain was calculated as in [75]. The distance between OH atoms of Tyr151, corresponding to the maximum constriction of the pore, were calculated between pairs of residues in opposite M3 helices in the connexon and averaged over the three possible opposite pairs. Molecular drawings were performed with VMD [76].

### Comparison with Electron Density map

The Cα scaffolds of our final model and that of ref. [11] were fitted against the electron density map, kindly provided by Julio Kovacs, using Situs 2.3 ([77], http://situs.biomachina.org/), a package for fitting high resolution models into low resolution maps. Using this computational tool one is able to map the tridimensional determinants of both the map and the model to a small number of points (*codebook vectors*). The two sets of codebook vectors are superimposed to find the best fit, after an exhaustive search in the translational and rotational degrees of freedom. The rigid docking fit was performed using 12 codebook vectors for both Cα models as well as for the electron density map.

## Supporting Information

Text S1 (0.03 MB DOC)

Figure S1 Reconstruction and structural relaxation of the KcsA potassium channel from its Ca atoms. Global RMS deviations vs. time calculated using the crystallographic structure as reference. The four shoulders in the curve correspond to the time points in which constraints are applied (20 ps) or increased (120, 220 and 320 ps respectively). The inset shows the RMS fluctuations calculated for each residue. The four polypeptide chains are indicated by different colors. The thick lines near the abscissa indicate the helical elements in the structure. The molecular drawings show least square superposition between the crystallographic (starting) structure and the reconstructed model after 20 ps, 120 ps, 220 ps and 320 ps of simulation, respectively.
(4.34 MB TIF)

Figure S2 Figures S2a and S2c to S2h. Location and interactions of residues along TM2 taken from the averaged structure of the whole connexon. Figure S2b. The exposed side chain of Trp77 is in contact with the membrane phospholipids, i.e., in good position to interact with cholesterol molecules (not present in the simulation). Shown is a representative snapshot from the MD trajectory.
(8.19 MB TIF)
