# Supplementary material for: A Fully Atomistic Model of the Cx32 Connexon
Source: PLoS One. 2008 Jul 2;3(7):e2614. doi: 10.1371/journal.pone.0002614 (PMC2481295; doi:10.1371/journal.pone.0002614)
Supplement: Text S1 — (0.03 MB DOC) [file pone.0002614.s001.doc]

**Supplementary Information**

# A fully atomistic model of the Cx32 connexon

**Sergio Pantano^1, 2, 3^ Francesco Zonta^2,3^ and Fabio Mammano^2, 3, 4^**

*^1^ Institut Pasteur of Montevideo*

*^2^ Venetian Institute of Molecular Medicine (VIMM)*

*^3^ Consorzio Nazionale Interuniversitario per le Scienze Fisiche della Materia (CNISM)*

*^4^ Dipartimento di Fisica “G.Galilei”, Università di Padova, Italy*

Structural determination of membrane channels constitutes one of the greatest advances of modern crystallography. Knowledge of the atomic detailed structure of several membrane channels have significantly contributed to the understanding on ions permeation and homeostasis. Nevertheless, achieving atomic detail resolution of membrane proteins is still a very challenging task and often only low-resolution models are obtained from X-ray crystallography and electron microscopy studies. In this context theoretical tools as modeling and molecular dynamics (MD) simulations have proven their value enriching the description of fundamental processes related to ionic channels such as ion binding and selectivity, hydration, etc. [34]. Furthermore, simulations offer a reliable alternative to evaluate the structural and functional roles of key amino acids and their mutants helping also to rationalize electrophysiological data.

In this contribution we present a methodology based in molecular modelling and MD techniques that allow gaining structural insights at amino acid-level starting from low-resolution structures containing only the Cα coordinates (see main text). In this supplementary section we present the results of a test case used to validate this procedure. We applied our method to the X-ray structure of the potassium channel from *Streptomyces Lividans* (KcsA) ([65] PDB entry code 1BL8). To this aim we kept only the Cα atoms present in the original PDB file removing all the rest. This set of Cα coordinates was used as input for the XLEAP module of AMBER 8 to add the lacking backbone and side chain atoms, similarly as we did for the Cα model of the connexon ([11] PDB entry code:1TXH). The resulting model underwent the procedure detailed in the Methods section of the main text.

The time evolution of the reconstructed model of KcsA channel is illustrated in Figure S1. The RMSD calculated for all the heavy atoms along the MD trajectory clearly shows an initial blow up of the structure during the first 20 ps of unconstrained simulation rising nearly 14 Å. This large distortion (Figure S1) is due to the internal clashes resulting from the side-chain reconstruction process. After this point, soft constraints are applied on the Cα and potassium atoms to pull them back to their original positions. In this way, the side chain’s atoms follow the Cα’s exploring conformations that are energetically compatible with the constraining force and with the restrictions imposed by the tertiary and quaternary structures of the protein environment. These constraints are increased after 100 ps and 200 ps, respectively resulting in a progressive reduction of the global RMSD that decrease below 2,5 Å. The root mean square fluctuations calculated over the last 300 ps shown in the inset of Figure S1 have a mean value of 2,1 (s.d. 0,7) Å these values are comparable with the results obtained by previous atomic-level molecular dynamics simulations on this system performed within an explicit solvent and membrane mimicking environment [19]. This agreement provides support to our model of the connexon. Therefore, although this model may not represent the actual arrangement of a connexon, the good agreement with a large number of biochemical data present in the literature suggest that the errors in the side chain assignation would remain within the limitation of state of the art simulations.

# Legends to Supplementary figures

**Figure S1**. Reconstruction and structural relaxation of the KcsA potassium channel from its Cα atoms. Global RMS deviations vs. time calculated using the crystallographic structure as reference. The four shoulders in the curve correspond to the time points in which constraints are applied (20 ps) or increased (120, 220 and 320 ps respectively). The inset shows the RMS fluctuations calculated for each residue. The four polypeptide chains are indicated by different colors. The thick lines near the abscissa indicate the helical elements in the structure. The molecular drawings show least square superposition between the crystallographic (starting) structure and the reconstructed model after 20 ps, 120 ps, 220 ps and 320 ps of simulation, respectively.

**Figure S2.** Figures S2a and S2c to S2h. Location and interactions of residues along TM2 taken from the averaged structure of the whole connexon. Figure S2b. The exposed side chain of Trp77 is in contact with the membrane phospholipids, i.e., in good position to interact with cholesterol molecules (not present in the simulation). Shown is a representative snapshot from the MD trajectory.
