# Supplementary figures and images for: A Fully Atomistic Model of the Cx32 Connexon
Source: PLoS One. 2008 Jul 2;3(7):e2614. doi: 10.1371/journal.pone.0002614 (PMC2481295; doi:10.1371/journal.pone.0002614)

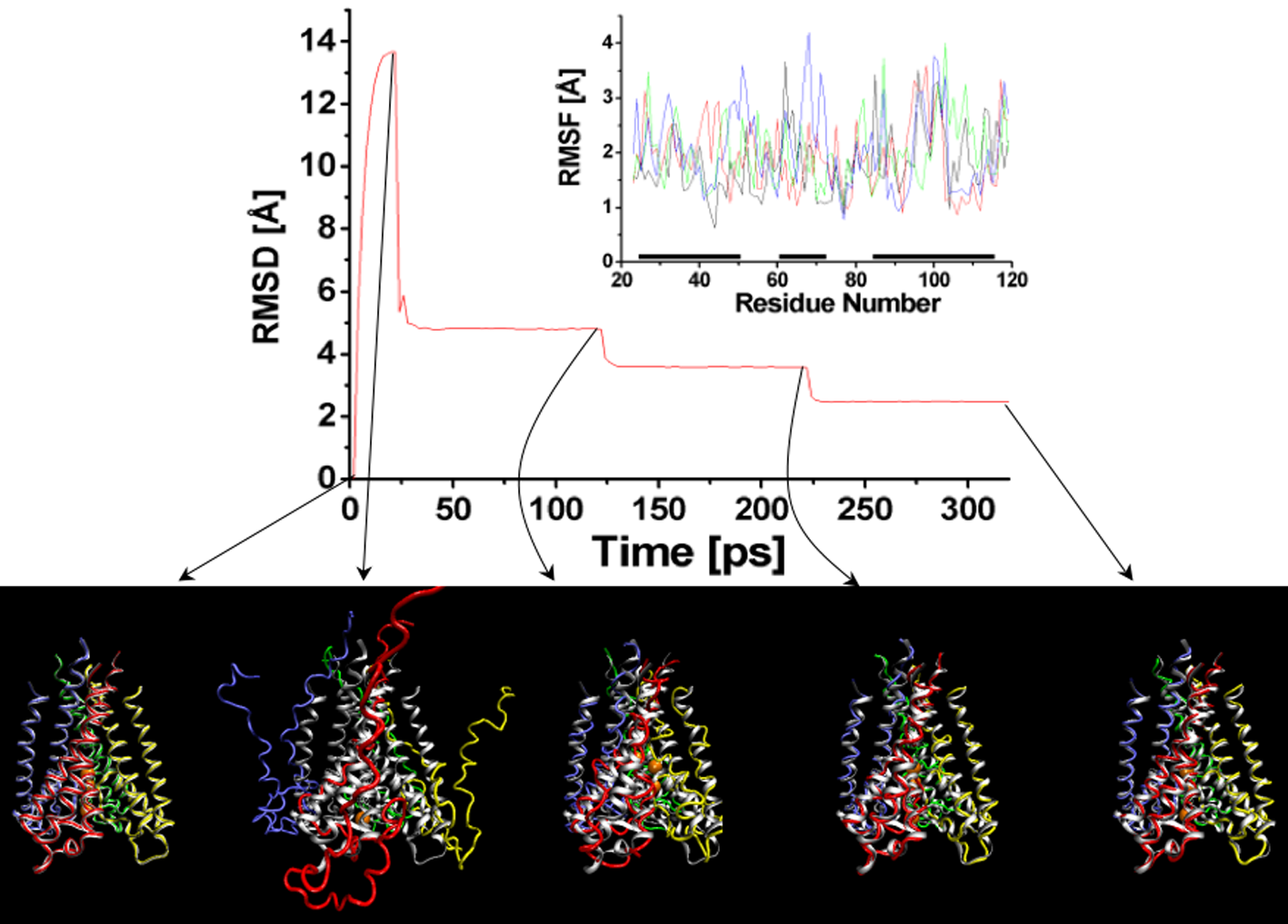

Supplement: Figure S1 — Reconstruction and structural relaxation of the KcsA potassium channel from its Ca atoms. Global RMS deviations vs. time calculated using the crystallographic structure as reference. The four shoulders in the curve correspond to the time points in which constraints are applied (20 ps) or increased (120, 220 and 320 ps respectively). The inset shows the RMS fluctuations calculated for each residue. The four polypeptide chains are indicated by different colors. The thick lines near the abscissa indicate the helical elements in the structure. The molecular drawings show least square superposition between the crystallographic (starting) structure and the reconstructed model after 20 ps, 120 ps, 220 ps and 320 ps of simulation, respectively. (4.34 MB TIF) [file pone.0002614.s002.tif]

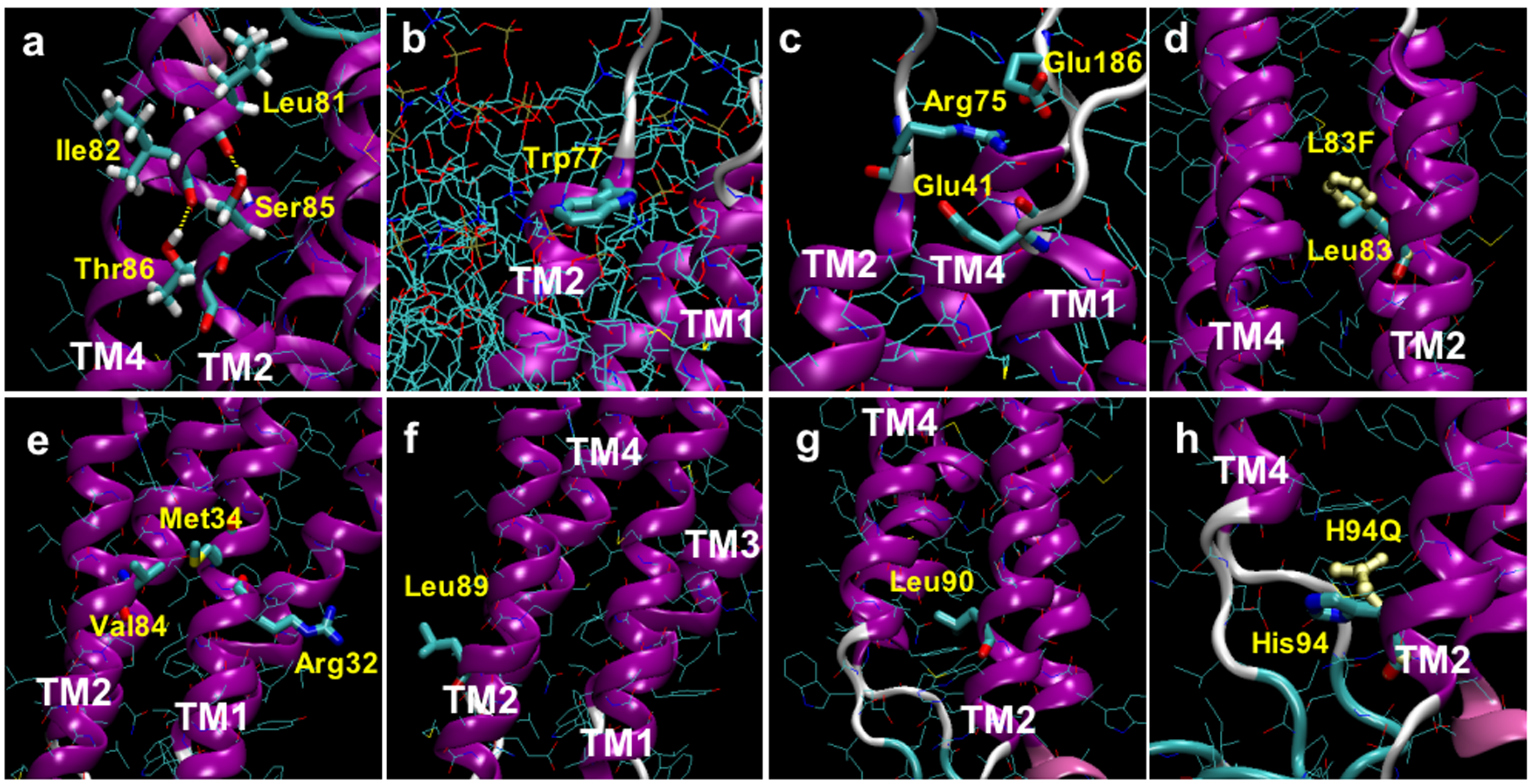

Supplement: Figure S2 — Figures S2a and S2c to S2h. Location and interactions of residues along TM2 taken from the averaged structure of the whole connexon. Figure S2b. The exposed side chain of Trp77 is in contact with the membrane phospholipids, i.e., in good position to interact with cholesterol molecules (not present in the simulation). Shown is a representative snapshot from the MD trajectory. (8.19 MB TIF) [file pone.0002614.s003.tif]
